# Early Thiopurines Versus Conventional Step-Care Therapy for Modifying the Disease Course of Early Crohn's Disease: A Tertiary Referral Center Cohort Study

**DOI:** 10.1097/MD.0000000000001148

**Published:** 2015-08-07

**Authors:** Yun Qiu, Bai-Li Chen, Ren Mao, Sheng-Hong Zhang, Yao He, Zhi-Rong Zeng, Min-Hu Chen

**Affiliations:** From the Department of Gastroenterology, The First Affiliated Hospital of Sun Yat-Sen University, Guangzhou, P.R. China.

## Abstract

The impact of thiopurines (TP) on the long-term outcome of early Crohn disease (CD) is still controversial. The present study designed as a comparison of conventional step-care to alternative treatment paradigms for disease progression.

This longitudinal cohort study examined the established CD patients from a university-based inflammatory bowel disease referral center. Outcomes of mucosal healing (MH), CD-related surgery or hospitalization, and clinical remission were compared based on timing of initiation of TP therapy. The cumulative incidence of events was estimated by Kaplan–Meier method.

One-hundred ninety patients with early CD were included. After a median follow-up of 57 months (interquartile range, 31.3–76.2), 29 patients undergone abdominal surgeries, 48 patients hospitalized, and 68 patients experienced clinical flares. A higher cumulative proportion of patients in the top-down (TD) group achieving MH than both the accelerated step-up (AC) group and conventional management (CM) group at month 36 (78.8% vs 39.9% and 42.2%, respectively; *P* = 0.001). There was a trend, albeit not significant, for an increased proportion of patients free of CD-related intestinal surgery in the TD group at month 60 (*P* = 0.16). However, among secondary outcomes, an early TP-based AC or TD strategy was not associated with improvement in clinical remission rates compared with a CM strategy at month 60 (*P* = 0.79). No significant difference was observed between early TP and CM for rates of MH, CD-related intestinal surgery or hospitalization, and clinical remission.

Both AC and CM strategy were minimally effective for disease modification. TD strategy has the potential of achieving higher rates MH. Our results support the TD strategy in patients with early CD at risk for a disabling course.

## INTRODUCTION

Crohn disease (CD) is a chronic, progressive, disabling, and destructive inflammatory disorder.^1^ Current guidelines advocate a step-up strategy to treatment, with the addition of more powerful therapies as the severity of disease or refractoriness to therapy increases. In this conventional step-care approach, anti-tumor necrosis factors (TNFs) are reserved for refractory or intolerance to conventional therapies. This approach has not been proven to slow or prevent bowel damage. In contrast to conventional step-up approach, the proactive top-down (TD) regimen or an accelerated step-up (AC) algorithm advocates biological and immunomodulator therapy at an early stage, shortly after diagnosis of CD. Whether such treatment paradigms outperformed the conventional step-care for the endpoints of disease progression and bowel damage remains a matter of active debate.

Accumulating evidence has pointed to the lack of efficacy of early use of thiopurines (TP) for achieving CFREM. Cosnes et al^2^ and Panes et al^3^ published their trials on early use of azathioprine (AZA) in adult CD for achievement of corticosteroid-free remission (CFREM). Both trials concluded that an AZA-based AD strategy was not associated with improvement in CD remission rates compared to a conventional step-up treatment strategy. Instead, trials with targets addressing bowel structure damage, evident as delay of disease progression or avoidance of bowel surgery, can be achieved by TP started early in CD course. Preliminary data from the Randomized Evaluation of an Algorithm for Crohn's Treatment (REACT) trial,^4^ which assigned patients with CD to either a conventional step-care or an AC algorithm with early use of combined antimetabolite/ADA therapy, also demonstrated marginally higher proportion of patients with AC in clinical remission at 12 months. However, after 24 months, a significant reduction in rates for complication, surgeries, and hospitalizations was observed using the AC approach.^4^ It is hence worth considering a treat-to-target strategy of TP treatment in CD.

Thus the present study designed as a comparison of conventional step-care to alternative treatment paradigms for the endpoints of bowel damage in a relatively large cohort of patients with CD.

## METHODS

### Patients and Design

This is an observational study where a prospectively designed standardized follow-up schedule was used for all patients.

All consecutive patients with a diagnosis of CD who received AZA/6-mercaptopurine (6-MP) treatment at the gastroenterology outpatient clinic of the first affiliated hospital of Sun Yat-Sen University between 2003 and 2013 were included in this study. Diagnoses of CD were established according to the criteria of Lennard-Jones,^5^ disease phenotype was determined according to the Montreal classification.^6^

Criteria for inclusion in the study were patients ages between 18 and 80 years old; early CD according to Paris definition^7^; at high risk for disabling disease^8^; continuous treatment with AZA/6-MP for at least 17 weeks.^9^

Exclusion criteria of this study: patients with an immediate need for surgery, or with contraindication to TP.

The study protocol was approved by the clinical research ethics committee of the first affiliated hospital of Sun Yat-Sen University.

### Definitions

Early CD is defined by disease duration ≤18 months and no previous use of disease-modifying agents.^7^ Patients were categorized into 1 of 3 groups based on the 3 main strategies which have been widely proposed to treat CD: conventional step-up therapy, early TD strategy, and the AC strategy. The AC strategy was defined as AZA/6-MP prescribed concomitantly with the first course of corticosteroids. Early TD strategy was referred to the combination of AZA/6-MP with anti-TNFs. The conventional management (CM) was classified as TP in condition of corticosteroid dependency or refractoriness, chronic active disease with frequent flares, or development of severe perianal disease.^2^ Patients applied the former 2 strategies were consisted the early TP group.

### Treatment Schedules: Dosing and Duration

According to the major available guidelines,^10–12^ AZA dose was targeted at 2.0 to 2.5 mg/kg body weight and 6-MP at 1.0 to 1.5 mg/kg body weight to achieve the therapeutic window of 250 to 400 pmol/8 × 10^8^ erythrocyte by regular monitoring the 6-thioguanine nucleotides(6-TGN) concentrations.^13^

### Endoscopic Documentation

All endoscopic procedures were performed by skilled endoscopists with standard protocol. The endoscopy reports were recorded in the patients’ pro forma questionnaire sheet and also saved as a digital version in the endoscopy registry. Score was assessed retrospectively by these 2 experts (B-LC and YH) according to the saved endoscopy images. Of note, the subsequent endoscopic assessments were usually a priori planned within 6 months by the treating physician to assess response to therapy.

### Clinical Follow-Up

All clinical follow-up information documented in the medical files of the patients was reassessed by 2 experienced gastroenterologists (B-LC, YH). A predetermined structured datasheet was used to collect data from the medical files, the inflammatory bowel disease (IBD) register, and the endoscopic register, which including age, gender, CD classification, smoking history, symptoms at presentation, presence of extra-intestinal manifestations, perianal lesions, endoscopic appearance, disease duration, TP initiation dates and dosage, and co-medication and start dates (eg, steroids, immunosuppressants, anti-TNFs use), need for bowel surgery or hospitalization. The laboratory values of hematology, immunology, chemistry, and microbiology were collected. The number of patients with mucosal healing (MH) was also recorded at the time of each endoscopic procedure.

### Outcomes

The primary efficacy outcome was the proportion of the achievement of MH and CD-related hospitalization, intestinal surgery at the successive visits throughout patient follow-up. MH was defined as in the study by Schnitzler et al^14^ in which complete MH was described as “the absence of ulcerations at follow-up endoscopy in patients who had ulcerations present at baseline ileocolonoscopy.” Surgery was defined as any intra-abdominal surgical procedure for active CD. CD-related hospitalizations were defined as those resulting from adverse events (AE) or CD-related treatment or complications.^15^

Prespecified secondary outcomes were the proportion of patients remaining in CFREM at the successive visits. CFREM was defined as the absence of flare, with no corticosteroid or anti-TNF use. Flare was defined by the Crohn's Disease Activity Index (CDAI) score >150 or an increase in CDAI of ≥70 points. Any new symptom or sign, any significant laboratory abnormality, or worsening of a preexisting condition or abnormality that occurred after initiation of TP was considered an AE.

When patients were lost to follow-up, the last date was taken to calculate the duration of TP therapy following guidelines of intention-to-treat.

### Statistical Analysis

Demographic and clinical parameters were described using medians with interquartile range (IQR) for continuous data and percentages for discrete data. Fisher's exact test and χ^2^ tests were used to compare the nonparametric categorical data between groups, and analysis of variance for continuous parameters. Cumulative probabilities of remaining free of events (perianal surgery, intestinal resection, and hospitalization) were calculated using the Kaplan–Meier method and compared with the log-rank test. Time-to-event analysis was performed with the Kaplan–Meier curve.

The SPSS 16.0 software (SPSS, Chicago, IL) were used to conduct all statistical analyses. For all tests, the *P* value for statistical significance is defined as *P* < 0.05.

## RESULTS

### Demographic Characteristics

The baseline characteristics of the 190 patients with early CD (129 male; median age, 26.9 years [IQR, 19.6–33.2 years]; median duration, 3 months [IQR, 1.3–6.8 months]) that we evaluated were shown in Table 1.

**TABLE 1:**
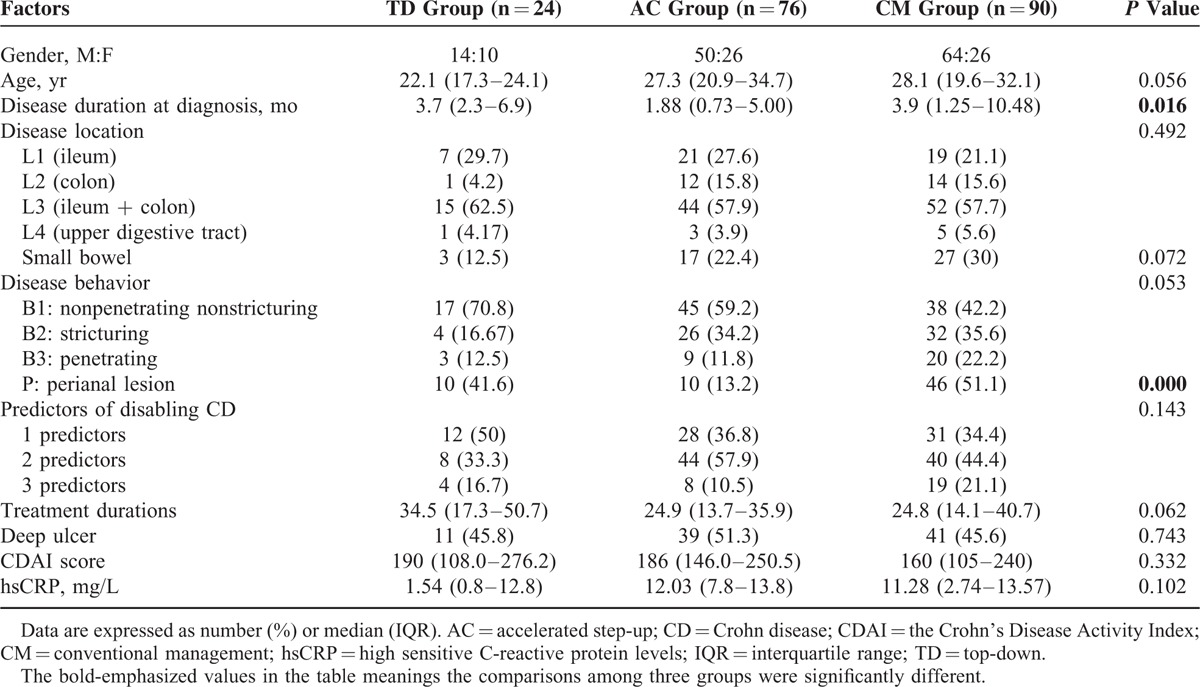
Comparison of Baseline Characteristics

**FIGURE 1:**
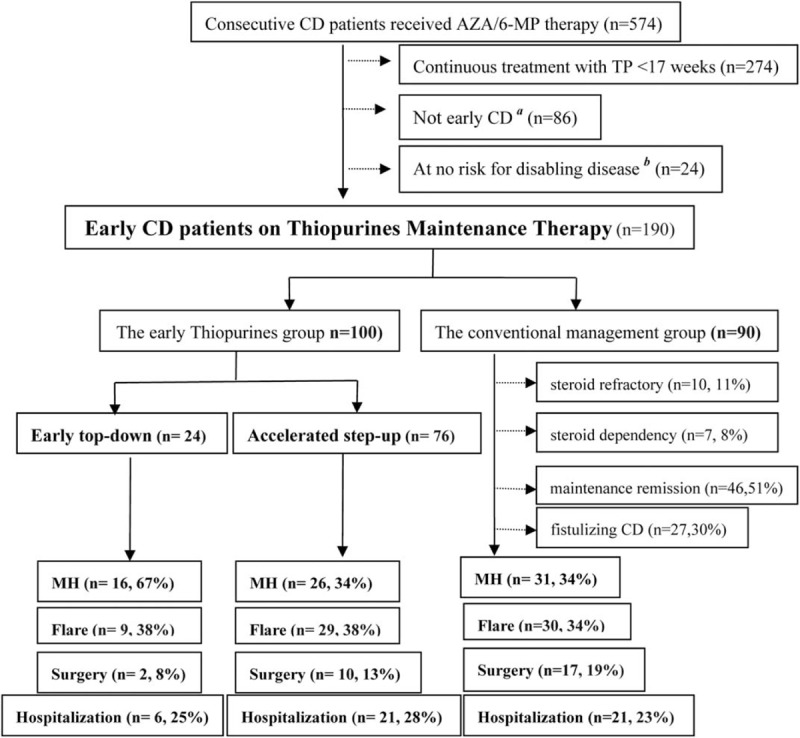
Study population. ^a^Early Crohn disease is defined by disease duration ≤18 months, no previous use of disease-modifying agents.^7^^b^Defined as thiopurines only in cases of corticosteroid dependency, chronic active disease with frequent flares, poor response to corticosteroids, or development of severe perianal disease.^2^

These 190 patients were then divided into 3 homogeneous arms by 3 main strategies which have been widely proposed to treat CD: the TD group (referred to the combination of AZA/6-MP with anti-TNFs, n = 24), the AC group (referred to AZA/6-MP prescribed concomitantly with the first course of corticosteroids, n = 76), and the CM group (referred to TP only in cases of corticosteroid dependency or refractory or development of severe perianal disease, n = 90) (Figure 1). There was no significant difference regarding age, gender, prior disease outcomes before referral, IFX use, ESR, new steroid use, and paralleled medication except for disease duration (*P* = 0.016) and perianal lesion (*P* < 0.001, Table 1). These patients were followed-up for a median of 57 months (IQR, 31–76 months).

### Primary Efficacy End Point

#### Endoscopic Procedures and Outcomes

**FIGURE 2:**
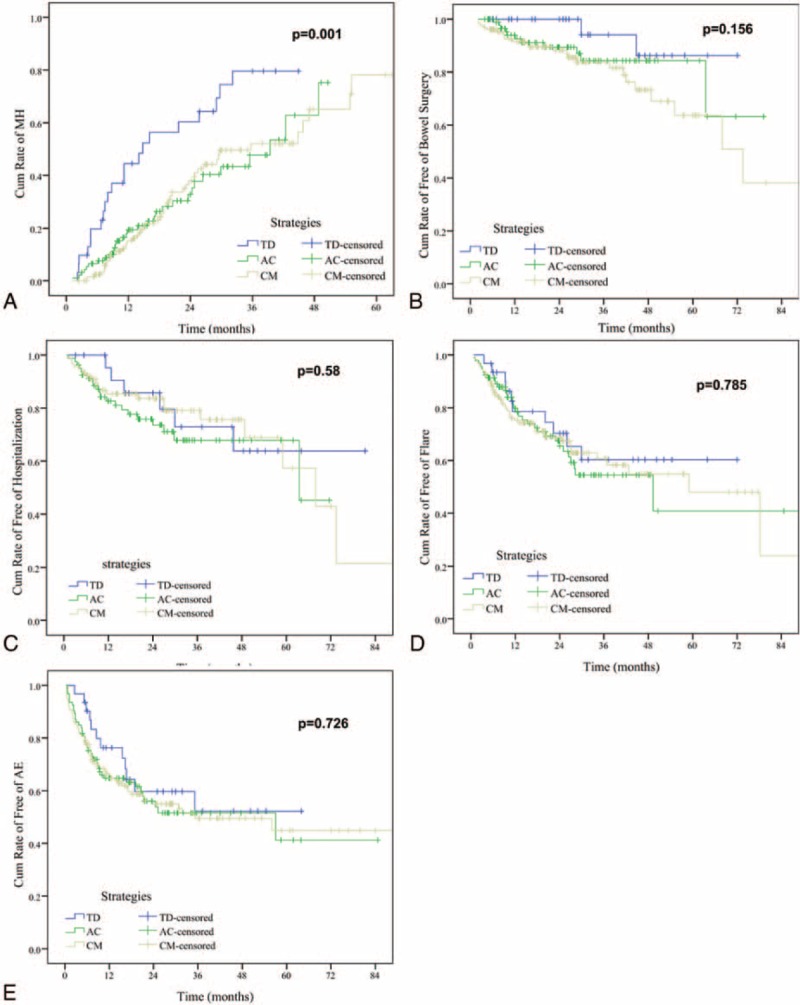
Kaplan–Meier plot showing probability of main events: (A) mucosal healing; (B) CD-related bowel surgery; (C) CD-related hospitalization; (D) flare; and (E) adverse events among patients assigned with different strategies. CD = Crohn disease.

MH was observed in 73 patients: 16 (16/24, 66.7%) were in the TD group, 26 (26/76, 34.2%) in the AC group, and 31 (31/90, 34.4%) in the CM group. A significant higher proportion of patients achieved MH in the TD group at month 36: 78.8 ± 9.4% in the TD group versus 39.9 ± 9.2% in the AC group, 42.2 ± 7.5% in the CM group (*P* < 0.01, Figure 2A).

#### Surgical Intervention and Hospitalizations

Due to persisting disease activity and strictures, 29 patients underwent intestinal resection: 2 (8.3%) were in the TD group, 10 (13.2%) in the AC group, and 17 (18.9%) in the CM group. There was a trend, albeit not significant, for an increased proportion of patients remaining free of intestinal surgery in the TD group at month 60: 83.2 ± 11% in the TD group versus 82.5 ± 6% in the AC group, 60.3 ± 9% in the CM group (*P* = 0.16, Figure 2B).

Hospitalization was observed in 48 patients: 6 were in the TD group, 21 in the AC group, and 21 in the CM group. There was no difference regarding the cumulative proportion of patients remaining free of hospitalization among the 3 groups at month 60: 63.9 ± 12.6% in the TD group versus 67.8 ± 6.4% in the AC group, 57.4 ± 12.6% in the CM group (*P* = 0.58, Figure 2C).

#### Secondary Efficacy End Points

There was a total 68 disease-flare attack within a median time 49.3 months (IQR, 28.6–70.4 months). Of the 68 patients experienced flare, 9 (37.5%) were in the TD group, 29 (38.2%) in the AC group, and 30 (33.3%) in the CM group. There was no significant difference regarding the cumulative proportion of patients in CFREM between the 3 groups at month 60: 55.4 ± 11.4% in the TD group, 24.9 ± 18.1% in the AC group, and 42.7 ± 11.5% in the CM group (*P* = 0.79, Figure 2D).

#### Toxicity

AEs were observed in 80 patients: 10 (41.6%) were in the TD group, 31 (40.8%) in the AC group, and 39 (43.3%) in the CM group. No difference regarding the cumulative proportion of patients free of AEs between the 3 groups at month 60: 51.1 ± 11.0% in the TD group versus 40.3 ± 13.0% in the AC group, 48.2 ± 7.0% in the CM group (*P* = 0.73, Figure 2E).

Most common AEs included myelotoxicity (13.2%), infections (9.5%), arthralgia (8.9%), flu-like symptoms (7.9%), and GI reactions (7.4%) were observed in the present study. The documented incidence of infections (*P* = 0.67) and arthralgia (*P* = 0.51) were comparable between the 3 groups. However, the incidence of leucopoenia was significantly lower in the CM group (*P* = 0.01).

Overall 21% of patients with CD discontinued therapy in our study. A discontinuation rate due to side effects was up to 11.6%, followed by refractoriness (4.7%) or patient's request (3.7%). And the incidence of withdraw were comparable among 3 groups except for that the myelosuppression caused withdraw was more frequently occurred in the TD group.

#### Early TP Compared With CM

**FIGURE 3:**
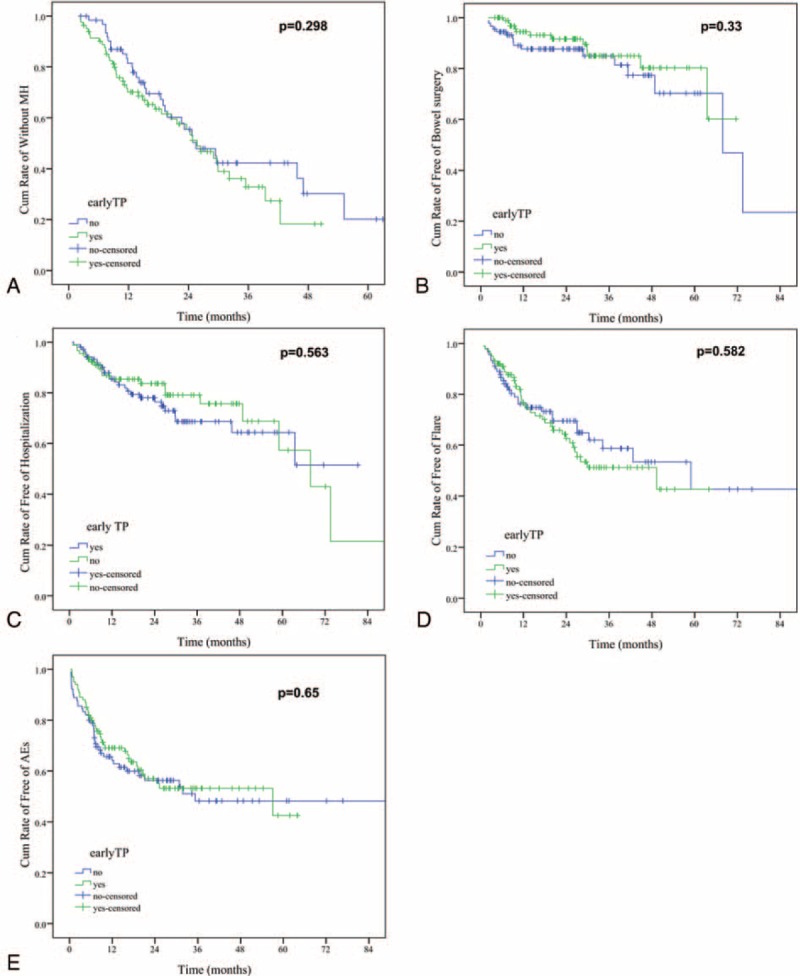
Kaplan–Meier plot showing probability of main events: (A) mucosal healing; (B) CD-related bowel surgery; (C) CD-related hospitalization; (D) flare; and (E) adverse events between early TP and conventional TP group. CD = Crohn disease; TP = thiopurines.

The former 2 strategies (TD and AC strategies) consisted the early TP group (n = 100). Considering both the primary and secondary endpoints, no significant difference was found between early TP group and conventional TP group (Figure 3).

## DISCUSSION

The present study focused on the long-term follow-up evaluation of 3 main strategies of TP treatment in early CD at risk for a disabling course. The efficacy of TP for maintenance of remission in patients with CD, albeit modest, is well established when used at the appropriate dosage and duration (a minimum of 17 weeks).^16^ In order to prevent progression to a complicated phenotype, it is of great importance to intervene in an early stage of the disease when irreversible damage has not yet occurred,^17,18^ the so-called “window of opportunity.”^19^

Recently, both the AZTEC study^3^ and the GETAID study^2^ failed to reproduce the efficacy of AZA in newly diagnosed adult CD, with the conclusion that AZA-based AC approach was no more effective for achieving clinical remission compared to a CM approach. However, both patient selection and the definition of the primary end point^20^ should be taken into account when interpreting the data.^21^ Importantly, nearly 30% of patients with complicated disease phenotype at diagnosis were excluded by definition from both AZTEC^3^ and RAPID trials.^2^

In the present study, only early CD defined as a disease duration of <18 months from diagnosis and also naïve to disease-modifying agents according to the Paris definition^7^ were included. TP and/or anti-TNFα were induced within a median time of 3 months (95% confidence interval 1.3–6.8) from diagnosis. One-seventh (14.7%) patients had undergone bowel complication at referral. In this selective cohort, we again failed to demonstrated early AZA/6-MP therapy (either TD or AC strategy) was more effective than CM for maintaining CFREM (*P* = 0.79).

Evidence is accumulating about the limitations of CDAI. Recently, a post hoc analysis of the SONIC trial further confirmed the discrepancy between clinical symptoms and objective findings of endoscopic inflammation,^22^ with an overall accuracy of clinical symptoms, relative to endoscopic inflammation of only 56%. Reliance merely on symptoms to guide treatment may be not an optimal management strategy.

MH has been received increasing attention based on observations that treatment aiming at clinical symptoms resolution alone does not reduce or prevent long-term bowel damage in patients with CD.^14,23–25^ MH as the treatment goal is being increasingly considered in patients with CD. Early introduction of TNF antagonists in the course of the disease, particularly in combination with immunosuppressives, is 1 strategy for improving MH, as shown in the SONIC and step-up/TD trials.^26,27^ Combination therapy with TP and infliximab could reduce anti-infliximab antibodies and approximately doubles the serum level of infliximab,^26,28^ which may lead to optimized clinical outcomes to either agent alone in both ulcerative colitis and CD.^26,29^ Whereas AZA monotherapy is minimally effective as a disease-modification agent in CD.^30^ In our cohort, there was a significant greater proportion of patients achieving MH in the early TP group (*P* = 0.001). The well-established evidence support that maintenance of MH is predictive of other desired disease-modification benefits such as prevention of bowel damage. The present study demonstrated a trend, albeit not significant, for an increased proportion of patients remaining free of intestinal surgery in the early TD groups at 5 years: 83.2% in the TD group versus 60.3% in the CM group (*P* = 0.16). As surgery is a relatively uncommon event in the first postdiagnostic 5 years in CD, it is possible a larger cohort with a longer follow-up would reveal differences between different strategies over time. As demonstrated in the REACT trial,^4^ a significant reduction in rates for complication, surgeries, and hospitalizations was observed using the AC approach after 24 months instead of 12 months. Thus, although due to the relative small group sample size, and low incidence rates of hospitalization and intestinal surgery, we did not demonstrate such benefits in the present cohort, it is promising such benefits exist due to its nature of MH on disease modification.

Safety issues should also be taken into account when initiating TD strategy. TP, when used in combination with anti-TNF agents, may be associated with an increased risk of opportunistic infections in patients with IBD.^31^ In the present study, safety was identical throughout the 3 groups (*P* = 0.89) during the 5 years’ follow-up. The rates of myelotoxicity (13.2%), infections (9.5%), arthralgia (8.9%), flu-like symptoms (7.9%), and GI reactions (7.4%) observed in the present study did not significantly differ from literature data.^32–35^ Overall 21% of patients with CD discontinued TP therapy mainly due to side effects which made up to 11.6% of our study population. In previous studies, TP discontinuation rate due to side effects varies from 5–6%^35^ to 30%.^36^ And the incidence of withdraw were comparable among 3 groups except for that withdraw due to myelosuppression was more frequently occurred in the TD group, possibly due to the synergetic effect of AZA and anti-TNFs.

This study had several limitations. First, the retrospective design could induce a bias of patient selection and information gathering. However, most of the data were collected prospectively and made these biases minimal. Second, tertiary-center referral bias was also likely to have influenced the outcomes considering the data source from a referral teaching hospital. Third, compliance to TP treatment was not assessed. But the regular check of 6-TGN concentration^37^ within the target therapeutic window suggested good compliance of our cohort. Fourth, the lack of a validated endoscopic scoring system was an important limitation. Finally, the lack of a uniformed tool to evaluate bowel damage is an important limitation. The development of novel tools, such as the Lemann score,^7^ will further our understanding of the potency of different strategies of TP in changing the natural course of CD.

In our single-center experience, both the AC and CM strategies are minimally effective for disease modification. TD strategy with potential of achieving higher rates MH, thus our results support the TD strategy in adult patients with early CD at risk for a disabling course.
